# Pelvic Exenteration for the Treatment of Locally Advanced Vulvar Cancer in South West Wales

**DOI:** 10.3390/cancers14071767

**Published:** 2022-03-31

**Authors:** Ganiy Opeyemi Abdulrahman, Nagindra Das, Thipparajapura V. Chandrasekaran, Umesh Khot, Peter J. Drew, Pradeep Bose, Jessica N. Vet, Nasima Tofazzal, Shaun Roberts, Kerryn Lutchman Singh

**Affiliations:** 1 Swansea Gynaecological Oncology Centre, Department of Obstetrics and Gynaecology, Swansea Bay University Health Board, Swansea SA2 8QA, UK; ganiy.abdulrahman@nhs.net (G.O.A.); nagindra.das@wales.nhs.uk (N.D.); jessica.vet@wales.nhs.uk (J.N.V.); 2 Swansea University Medical School, Swansea University, Swansea SA2 8PP, UK; 3 Department of Gastrointestinal and Colorectal Surgery, Swansea Bay University Health Board, Swansea SA6 6NL, UK; chandra.sekaran@wales.nhs.uk (T.V.C.); umesh.khot@wales.nhs.uk (U.K.); 4 Welsh Centre for Burns and Plastic Surgery, Swansea Bay University Health Board, Swansea SA6 6NL, UK; peter.drew@wales.nhs.uk; 5 Department of Urology, Swansea Bay University Health Board, Swansea SA6 6NL, UK; pradeep.bose@wales.nhs.uk; 6 Department of Cellular Pathology, Swansea Bay University Health Board, Swansea SA2 8QA, UK; nasima.tofazzal@wales.nhs.uk (N.T.); shaun.roberts@wales.nhs.uk (S.R.)

**Keywords:** vulvar cancer, pelvic exenteration, survival, chemoradiation, multidisciplinary, Wales

## Abstract

**Simple Summary:**

There is no consensus on the optimal treatment strategy for locally advanced vulvar cancer. In this paper, we aimed to highlight the outcomes from pelvic exenteration in our centre. Here, we not only demonstrated the role of pelvic exenteration in the treatment of locally advanced vulvar cancer, but we also revealed modifiable and non-modifiable factors that contribute to outcomes. Furthermore, we highlighted that tumours that are less than 40 mm diameter do not usually require flap reconstruction while tumours of 40 mm diameter or greater would often require flap reconstruction—a finding that we believe has not been previously reported in the literature. However, we recognise that research must continue into treatment options that limit the radicality of surgical resection, although such alternative approaches must offer comparable survival advantages.

**Abstract:**

The treatment of locally advanced vulvar carcinoma (LAVC) represents a major challenge. We investigated the role of pelvic exenteration as a treatment of LAVC. Women who underwent pelvic exenteration for primary and recurrent LAVC in our centre between 2001 and 2019 were included. Among the 19 women included during the study period, 14 women (73.7%) had primary LAVC while 5 women (26.3%) had recurrent disease. Surgical resection margins were microscopically clear (R0) in 94.7% of patients—14/14 undergoing primary treatment and 4/5 undergoing treatment for recurrent disease. Complete closure of the wound was achieved in 100% of women, with no wound left to heal by secondary intention. Tumour size was a predictor of requiring myocutaneous flap reconstruction, with all tumours less than 40 mm undergoing primary closure, while almost all tumours 40 mm diameter or greater (14/15 women) required flap reconstruction (*p* = 0.001). The 30-day major morbidity rate was 42% and there was no perioperative death. The mean overall survival was 144.8 months (2–206 months), with 1-, 2- and 5-year survival rates of 89.5%, 75.1% and 66.7%, respectively. In our centre, a primary surgical approach to the management of LAVC has resulted in good survival outcomes with acceptable morbidity rates.

## 1. Introduction

Vulvar cancer is a rare gynaecological malignancy with an incidence of 3.7 per 100,000 women in the UK [1]. Although a disease that mostly affects the elderly population, the incidence of vulvar cancer is increasing in young women presumably because of the effect of increasing human papillomavirus infection [1]. Unfortunately, vulvar cancer is associated with delay in both presentation and diagnosis with approximately 40% of women presenting with advanced disease (FIGO III or IV) [2,3]. Hence, the management of these women presents a major therapeutic challenge.

Whilst pelvic exenteration was the mainstay of treatment decades ago, various alternative treatment modalities with primary chemoradiation and neoadjuvant chemoradiotherapy followed by radical surgical excision have emerged in clinical practice [4,5]. However, there are no standardised protocols for chemoradiation in the management of locally advanced vulvar cancer. A Cochrane review identified four different chemotherapeutic schedules along with varied techniques for radiotherapy, involving different dose fractionations, treatment fields and targets [6].

The only randomised controlled study on the treatment of advanced vulvar carcinoma concluded that there was a high complication rate with neoadjuvant chemoradiation, with no significant advantage when compared to primary surgery [7]. Despite this, there has been no randomised controlled trial comparing primary surgery with primary chemoradiotherapy. Retrospective studies are variable in outcomes, with one study showing that whilst chemoradiotherapy and surgery can be utilised effectively as primary treatment depending on patient-specific factors at presentation, there was no survival advantage between the two modalities [8].

Locally advanced vulvar cancer is either close to, or overtly involves neighbouring organs such as the vagina, urethra, vesical mucosa, anus and/or rectum [9]. Surgical resection of the tumour is usually the treatment of choice, especially when it can avoid sphincter damage leading to urinary and/or faecal incontinence and it is often preferable to use primary radiotherapy to prevent the need for a stoma [10]. Only a few studies have specifically addressed the predictive factors and outcomes of pelvic exenteration for locally advanced vulvar cancer with five-year survival rates reported as 25–62% [11,12,13]. Whilst primary chemoradiotherapy appears to be gaining wider acceptance for these patients, the lack of a standardised protocol means that the role of exenterative surgery remains pertinent [10,14].

The aims of this study were to evaluate the outcomes of women who received treatment with pelvic exenteration for locally advanced primary or recurrent vulvar cancer in our centre and to analyse factors that influenced prognosis.

## 2. Materials and Methods

### 2.1. Study Design and Population

This was a retrospective analysis of women who underwent pelvic exenteration for locally advanced vulvar cancer at our centre between 2001 and 2019. Data capture for women undergoing surgery for vulvar cancer was facilitated by a prospectively maintained database at the Swansea Gynaecological Oncology Centre and theatre records. We also reviewed the oncological outcomes for women treated with primary or neoadjuvant radiotherapy/chemoradiotherapy for locally advanced vulvar cancer between 2011 and 2019 for comparison. Exenterative surgery was performed if the patient had proximal (upper 2/3) vaginal and/or anal involvement, and if the excision of the anus will result in adequate clearance. Patients requiring only lower third urethral excision were excluded from the study as these patients were managed with radical wide local excision alone. This study was approved by the local clinical audit and effectiveness department.

### 2.2. Procedures

Following the identification of women who have had pelvic exenteration from our surgical database, we collected information on their hospital identifier. The demographics of the patients, multidisciplinary team discussion, clinic letters, operative notes, perioperative events and histology reports were reviewed. In cases where relevant histological features were not reported, our multidisciplinary team pathologists reassessed the specimens and histological slides for verification of the dataset. Perioperative mortality was defined as any death that occurred in or out of the hospital within 30 days of surgery. Surgical morbidity was categorised based on the Clavien–Dindo classification [15]. Major morbidity was defined as Clavien–Dindo ≥3.

### 2.3. Statistical Analysis

All analyses were performed on an intention-to-treat basis. Descriptive statistics were used to summarise demographic information. Mean and median values were calculated where appropriate. Statistical comparisons were conducted using Pearson’s Chi Square test to analyse categorical variables as appropriate. Individuals were followed from the time of surgery until death occurrence or study end date at which point survival data were censored (31 May 2020). Survival was estimated using Kaplan–Meier analysis. Statistical analyses were performed using IBM SPSS 21.0 (SPSS Inc., Chicago, IL, USA) and statistical significance was reported using a two-tailed α = 0.05.

## 3. Results

### 3.1. Characteristics of Patients and Outcomes of Treatment for Locally Advanced Vulvar Cancer

Between April 2001 and December 2019, a total of 19 patients underwent exenterative surgery for locally advanced vulvar cancer, 14 (73.7%) as primary treatment and 5 (26.3%) as treatment for recurrent disease. Data from 2005 to present indicated that, on average, we treat 20 new patients with vulvar cancer annually, with approximately 2 patients per year having locally advanced disease. During the period of 2011 to 2019, 12 patients underwent primary radiotherapy treatment for locally advanced vulvar cancer. The radiation protocol during this period varied, ranging from 20 Gy in 5 fractions to 45 Gy in 25 fractions. The mean survival in this group was 9.5 months (range 3.6–15.4 months). The overall 1-, 2- and 5-year survival rates for women undergoing primary radiotherapy were 41.7%, 8.3%, and 0%, respectively.

Patient demographics are summarised in Table 1. Mean age at diagnosis was 65.2 years (47–81 years) and mean body mass index was 30.3 kg/m^2^ (range 19.9–40 kg/m^2^), while median length of follow up for the entire cohort was 69 months (2–206 months).

The mean tumour diameter was 52.8 mm (18–100 mm) and the only histological type was squamous cell carcinoma, with most women (94.8%) having moderately to poorly differentiated cancers. Most patients (73.7%) had posterior pelvic exenteration while total pelvic exenteration was performed in five patients. Surgical resection margins were microscopically clear (R0) in 94.7% of patients—14/14 undergoing primary treatment and 4/5 undergoing treatment for recurrent disease. Macroscopic clearance of tumour was achieved in all patients.

Bilateral inguino-femoral (groin) lymph node dissection was performed in 13 patients (12/14 primary, 1/5 recurrent), with inguinal nodal metastatic disease seen in 9 patients (8/12 primary and 1/1 recurrent). One patient with recurrent disease had unilateral inguino-femoral (groin) lymph node dissection because of suspicious unilateral groin lymph node preoperatively and metastatic nodal disease was confirmed on histology. In one patient with recurrent disease, nodal debulking was performed followed by postoperative groin and pelvic radiotherapy. Three patients in the primary surgery group with multiple positive groin nodes and/or extracapsular spread received postoperative radiotherapy, mostly in the form of external beam radiation therapy (EBRT). Complete closure of the wound (including the pelvic and vulvar-perineal defect) was achieved in 100% of patients, with no wounds left to heal by secondary intention. Plastic reconstructive techniques were utilised for most patients (*n* = 13, 69.4%), although primary closure without the need for plastic reconstruction was achieved in almost a third of the women (*n* = 6, 31.6%).

The 30-day major morbidity rate was 42% and there was no perioperative mortality. The mean blood loss was 955 mL (150–5000 mL) and mean length of stay was 27 days (9–100 days). Both the mean blood loss and length of stay were significantly higher during our early experience with pelvic exenteration. The mean estimated blood loss was 2076 mL (500–5000 mL) pre-2011 while it was 395 mL (150–1000 mL) from 2011 to 2019. Similarly, the mean length of stay was 37.9 days (13–100 days) pre-2011, compared to 19.2 days (9–39 days) in the last 10 years. Tumour size was a predictor for requirement of myocutaneous flap reconstruction. All tumours less than 40 mm diameter did not require flap reconstruction while 14/15 women (93.3%) with tumours of 40 mm diameter or greater required flap reconstruction (*p* = 0.001). Myocutaneous flap morbidity (defined as any morbidity directly related to the myocutaneous flap) was a significant contributor to overall morbidity (*p* = 0.002) but there was no significant correlation between myocutaneous flap morbidity and length of stay. The mean overall survival for the entire cohort was 144.8 months (2–206 months), with 1-, 2- and 5-year survival rates of 89.5%, 75.1% and 66.7%, respectively.

### 3.2. Pelvic Exenteration for Primary Treatment of Locally Advanced Vulvar Cancer

The primary surgery group mostly consisted of women with FIGO stage IVA disease (*n* = 10, 71.4%). The remaining patients (*n* = 4, 28.6%) required part of the upper 2/3 of the vagina resected to achieve clear margins, two of whom were confirmed to have inguino-femoral metastases postoperatively and were classed as FIGO stages II and III (Table 1). All patients in this group had no prior radiotherapy treatment and opted for primary surgical treatment. The mean age of women was 62.2 years (47–81 years). In all women, microscopic clearance (R0) was achieved. A total of 12/14 women (85.7%) also underwent bilateral groin nodes dissection while two women declined bilateral groin nodes dissection in favour of radiotherapy and ultrasound surveillance.

**Figure 1:**
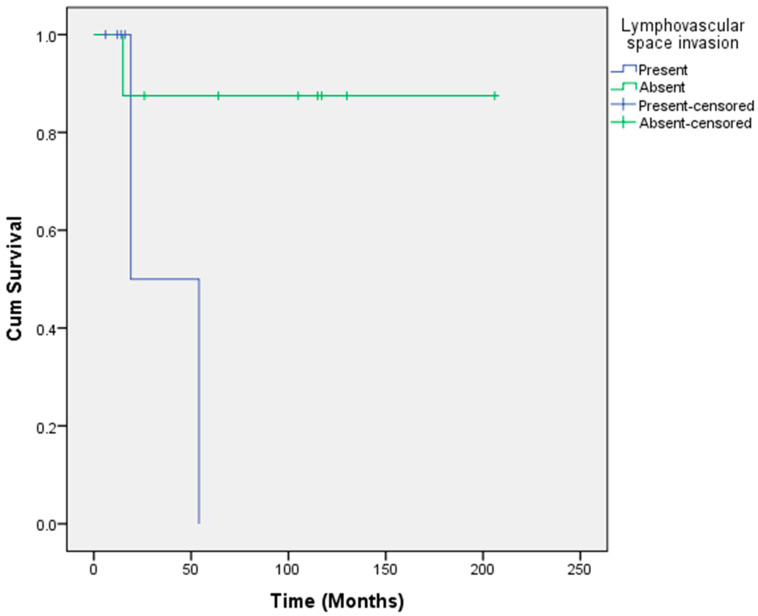
Lymphovascular space invasion was a predictor of survival in the primary surgery group with the presence of lymphovascular space invasion associated with worse prognosis (*p* = 0.05).

The mean overall survival for this group of women was 152.2 months (6–206 months). The mean overall survival for women with positive lymph nodes was 44.3 months (6–117 months), while node negative women had a mean overall survival of 88.1 months (15–206 months). There was only one woman in the node positive group with extracapsular spread. On multivariate analysis, survival was not significantly associated with age, lymph node status or tumour diameter. However, prognosis was significantly worse if lymphovascular space invasion was present in the primary tumour. The mean survival among women with lymphovascular space invasion was 36.5 months compared to 182.1 months when lymphovascular space invasion was absent (*p* = 0.05) (Figure 1).

**Figure 2:**
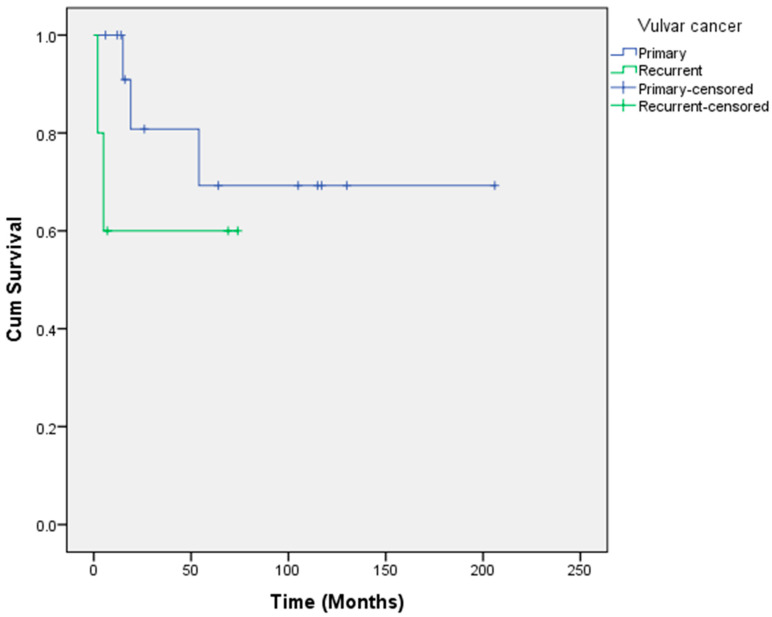
Kaplan–Meier estimates of survival for pelvic exenteration for primary and recurrent locally advanced vulvar cancer.

The mean overall survival among women with perineural invasion was 91.7 months in comparison to 153.2 months when perineural invasion was absent, but this was not statistically significant (*p* = 0.772). The overall 1-, 2- and 5-year survival rates were 100%, 80.8% and 69.3%, respectively (Figure 2). All patients who survived beyond five years in our study group were still alive at 10 years (Table 1).

### 3.3. Pelvic Exenteration for Recurrent Locally Advanced Vulvar Cancer

The recurrent group consisted of five patients, with two patients having received prior radiotherapy. The first patient had adjuvant radiotherapy to the vulva and both groins but had an advanced local relapse soon afterwards and underwent exenteration. Unfortunately, this patient suffered a colonic perforation two months after surgery (from nonmalignant pathology) and did not survive. The second patient was treated with radiotherapy to the vulva for a local relapse four years after initial treatment. This patient presented again with a locally advanced relapse 12 years after her radiotherapy treatment and is still alive six years following her exenterative procedure. The mean age of women in this group was 73.6 years (70–78 years). There was a microscopically positive histological margin (R1) in one woman, who had a large recurrence involving most of the posterior vulva, extending to the distal urethra and with a metastatic groin node. This patient was originally treated with a radical local excision and sentinel lymph node biopsy (which was negative) and did not receive adjuvant radiotherapy. We removed the distal urethra with the specimen and although margins were macroscopically clear, the proximal urethral margin contained a microscopic remnant (R1). The woman declined further surgery and went on to start postoperative radiotherapy. Unfortunately, the woman relapsed and died within 6 months of surgery. As a result, the 1-year survival rate in this group was 60% and this remained the case at 5 years (Figure 2). The mean overall survival was 45.8 months (2–74 months). There was no significant predictor of survival in this small group of women (Table 1).

## 4. Discussion

Our results confirm that pelvic exenteration is a valid and effective treatment modality for selected patients with primary and recurrent locally advanced vulvar cancer.

The overall morbidity and survival rates in our study are comparable to other reported studies [11,12,16]. Studies that have specifically addressed the predictive factors and outcomes of pelvic exenteration for locally advanced vulvar cancer are limited and there has been no previous study on pelvic exenteration for locally advanced vulvar cancer in Wales.

Our five-year survival rates of 69.3% in the primary group and 60% in the recurrence group compare favourably with the reported literature [12,13,16].

In our experience, prognosis in women with locally advanced vulvar cancer is dependent on modifiable and non-modifiable factors. Non-modifiable factors primarily reflect the biology of the tumour—tumour size, lymphovascular space invasion and perineural invasion. Modifiable factors primarily reflect exclusion of distant metastases, accurate assessment of resectability, and the availability of multidisciplinary surgical expertise to achieve adequate excision margins (R0) with acceptable morbidity. Positron emission tomography is increasingly being used to exclude distant metastases [17,18]. Following a change in the delivery of surgical services at our centre in 2011, which allowed all the pelvic surgical specialties to be co-located, we have noticed a trend towards improved R0 rate and reduced morbidity, especially in terms of blood loss and length of stay.

Our aim is to achieve en bloc delivery of all involved pelvic organs, starting with a midline abdominal entry to mobilise the pelvic organs and ending with the perineal resection. This surgical approach is supported by Rodriguwz-Bigas and Petrelli’s assertion that potential cure in patients with adjacent organ involvement should not be compromised by doing less than an en bloc resection [19]. We have developed this surgical expertise over the past 20 years amongst a dedicated team of gynaecological oncologists, colorectal surgeons, urologists and plastic surgeons. Where significant disruption of the pelvic floor and extensive skin loss are anticipated, we favour a myocutaneous flap. In our centre, the need for a myocutaneous flap is usually predictable with large lesions (≥4 cm), although we will also consider a myocutaneous flap in the setting of previous pelvic external beam radiotherapy.

In the report by O’Donnell and colleagues, complete primary closure of the vulvar wound was achieved in less than 40% of their cohort, with the remaining patients having their vulvar wound left partially or entirely open to heal by secondary intention [13]. None of the women in their study underwent reconstructive surgery [13]. This is in contrast with our cohort, where almost 70% of the women had reconstructive surgery. Our observation that virtually all tumours 40 mm or greater in diameter will require myocutaneous flap reconstruction indicates that when planning the surgery for such patients, it is important to ensure the availability of plastic surgical expertise.

If a myocutaneous flap is not required as part of a posterior exenteration, we have developed an inter-sphincteric approach. Originally developed for management of low rectal tumours, the intersphincteric approach is a surgical technique extending rectal resection into the intersphincteric space. This procedure is performed by a synchronous abdominoperineal approach with mesorectal excision and excision of the entire or part of the internal sphincter with removal of the rectal stump [20]. In our experience, this approach is preferred as rectal stump-related morbidities may involve local inflammatory changes ranging from minor phlegmon to pelvic sepsis with a reported rate of 6.9% [21]. It also avoids the need for a mucous fistula, which adds another ostomy for the patient. Furthermore, this approach maintains a greater proportion of the pelvic floor musculature, which facilitates primary closure. Primary skin closure is achieved with monocryl. Lesions that extend near the urethral meatus (within 1 cm) result in removal of the lower third of the urethra. In these women, a long-term catheter is left in situ for 6 weeks. Proximal (upper two-thirds) vaginal involvement in our cohort was seen in women with very advanced disease and this resulted in total pelvic exenteration as the disease was circumferential in nature involving both the anterior and posterior vagina. These patients required an ileal conduit in addition to an end colostomy.

In the primary treatment group, lymphovascular space invasion was a significant prognostic factor but this was not reflected in the recurrent treatment group, possibly due to the small number of women in this group. Lymphovascular space invasion has previously been identified as a predictor of survival in patients undergoing pelvic exenteration for various gynaecological malignancies [22,23,24]. The importance of perineural invasion as an independent predictor of poor prognosis has been previously identified [25,26]. In our primary group, there was a trend towards poor prognosis with the presence of perineural invasion, although this was not statistically significant.

The major criticisms of pelvic exenteration are the associated morbidities and the requirement for a stoma. Our 30-day major morbidity rate was 42% (mostly Clavien–Dindo IIIA) and there was no perioperative death. These rates are consistent with those reported in the literature [11,16,27].

The development of our surgical expertise in pelvic exenteration was partly because of the poor outcomes from primary radiotherapy for locally advanced vulvar cancer that we observed in our centre. Similar poor outcomes from primary radiotherapy for locally advanced vulvar cancer have also been reported in the literature. Published studies have reported low complete response rates to chemoradiation for locally advanced vulvar cancer, with complete response rates of just 50% or less and associated five-year survival rates of 30–50% [28,29,30,31,32]. The five-year overall survival rate of 66.7% reported in our study compares favourably with these studies. Furthermore, there is a significant variation in the protocols for chemoradiation in published studies [4,6,29,30,33]. This makes a standardised approach for chemoradiation in the management of locally advanced vulvar cancer a major challenge.

While most published studies on pelvic exenteration in gynaecological oncology included various gynaecological tumours, our study has provided detailed outcomes specific to vulvar cancer. However, a limitation of this study is that we have not reported on the quality-of-life outcomes for these women, including the impact of requiring a stoma.

Our results affirm that pelvic exenteration remains a valid treatment approach for patients with locally advanced vulvar cancer. Complete surgical excision, with good survival outcomes and acceptable morbidity, is achievable with a multidisciplinary surgical approach. It is therefore essential that pelvic exenteration is offered to women with locally advanced vulvar cancer only in centres with adequate case volumes where these multidisciplinary skills have been developed. Prognosis is dependent on modifiable and nonmodifiable factors. Treatment options that limit the radicality of the resection should continue to be explored as they offer the potential for avoidance of stomas and therefore a better quality of life for women. However, they must offer a comparable survival advantage and should be standardised.

## 5. Conclusions

In our centre, a tailored approach to primary radical surgical treatment of locally advanced vulvar cancer in a well-established multidisciplinary team has resulted in good survival outcomes with acceptable morbidity.

## Figures and Tables

**Table 1:** Baseline characteristics, procedures and outcomes.

	All	Primary	Recurrence
Patient Demographics			
Number of patients (*n*)	19	14	5
Mean age (years, range)	65.2 (47–81)	62.2 (47–81)	73.6 (70–78)
Mean BMI (*n* = 12, range)	30.3 (19.9–40)	30.4 (19.9–40)	29.8 (24.5–37)
American Society of Anesthesiologists (ASA) Physical Status (*n*/%)			
I	1 (5.3)	1 (7.1)	0 (0)
II	6 (31.6)	5 (35.7)	1 (20)
III	5 (26.3)	3 (21.4)	2 (40)
Missing	7 (36.8)	5 (35.7)	2 (40)
Surgical Outcomes			
Figo Stage (*n*,%)			
Stage II	2 (14.2)	2 (14.2)	N/A
Stage III	2 (14.2)	2 (14.2)	N/A
Stage IV	10 (71.4)	10 (71.4)	N/A
Histology (*n*/%)			
Squamous cell carcinoma	19 (100)	14 (100)	5 (100)
Differentiation (*n*/%)			
Well	1 (5.2)	0 (0)	1 (20)
Moderate	12 (63.2)	10 (71.4)	2 (40)
Poorly	6 (31.6)	4 (28.6)	2 (40)
MEAN TUMOUR DIAMETER (range, mm)	52.8 (18–100)	54.7 (18–100)	47.6 (32–60)
Resection Margin (*n*, %)			
Surgical R0 (microscopically negative)	18 (94.7)	14 (100)	4 (80)
Surgical R1 (microscopically remnant)	1 (5.33)	0 (0)	1 (20)
Surgical R2 (macroscopic remnant)	0 (0)	0 (0)	0 (0)
Nodal Status			
Inguinal lymph node metastases (*n*, %)	10 (71.4)	8 (66.7)	2 (100)
Lymphovascular space invasion present (*n*, %)	9 (47.4)	6 (42.9)	3 (60)
Perineural invasion present (*n*, %)	8 (42.1)	5 (35.7)	3 (60)
Lymphovascular space invasion + Perineural invasion present (*n*, %)	4 (21.1)	2 (14.3)	2 (40)
Node positive without extracapsular spread	8	7	1
Node positive with extracapsular spread	2	1	1
Node negative	4	4	0
Surgical Procedure (*n*, %)			
Posterior exenteration	14 (73.7)	11 (78.6)	3 (60)
Total exenteration	5 (26.3)	3 (21.4)	2 (40)
Ileal conduit	5 (26.3)	3 (21.4)	2 (40)
Reconstruction (*n*, %):			
Primary closure	6 (31.6)	4 (28.6)	2 (40)
Vertical Rectus Abdominis Myocutaneous Flap (VRAM)	5 (26.3)	3 (21.4)	2 (40)
Bilateral gracilis myocutaneous flap	2 (10.5)	2 (14.3)	0 (0)
VRAM + gracilis	3 (15.8)	2 (14.3)	1 (20)
Inferior gluteal artery myocutaneous (IGAM)	1 (5.3)	1 (7.1)	0 (0)
Fasciocutaneous flap	2 (10.5)	2 (14.3)	0 (0)
Perioperative Features			
Mean blood loss (mL, range)	667 (150–2180)	798 (200–2180)	338 (150–500)
Major morbidity {Clavien–Dindo Grade 3 and above, *n* (%)}	9 (47.4)	6 (42.9)	3 (60)
Mean length of stay (days)	20 (9–39)	19 (9–39)	24 (14–30)
30-day major morbidity rate (*n*, %)	8/19 (42.1%)	6/14 (42.9%)	2/5 (40%)
30-day mortality rate	0	0	0
Survival			
Overall survival (months, range)	144.8 (2–206)	152.2 (6–206)	45.8 (2–74)
% 1-year survival	89.5	100	60
% 5-year survival	66.7	69.3	60
% 10-year survival	66.7	69.3	Not reached yet
Overall survival when lymphovascular space invasion present (months)	44.1	36.5	51
Overall survival when lymphovascular space invasion absent (months)	166.5	182.1	35

## Data Availability

The datasets used and/or analysed during this study are available from the corresponding author on reasonable request.
